# Rapid Whole Genome Sequencing of Serotype K1 Hypervirulent *Klebsiella pneumoniae* from an Undocumented Chinese Migrant

**DOI:** 10.1155/2021/6638780

**Published:** 2021-04-28

**Authors:** C. K. Macleod, F. A. Khokhar, B. Warne, R. Wick, R. Butcher, B. Cassimon, P. Hayden, K. Holt, M. E. Török

**Affiliations:** ^1^Department of Clinical Research, London School of Hygiene & Tropical Medicine, London, UK; ^2^Medway Maritime Hospital, Gillingham, Kent, UK; ^3^Department of Medicine, University of Cambridge, Cambridge, UK; ^4^Department of Infectious Diseases, Central Clinical School, Monash University, Melbourne, Australia; ^5^Department of Infection Biology, London School of Hygiene & Tropical Medicine, London, UK; ^6^Public Health England, Clinical Microbiology and Public Health Laboratory, Cambridge, UK

## Abstract

**Background:**

Hypervirulent *Klebsiella pneumoniae* causes severe disseminated infections, typically with hepatic and central nervous system involvement including endophthalmitis. *Case Presentation*. We report a fatal case of an undocumented Chinese migrant in her 60s who presented to the emergency department with abdominal pain, lethargy, and headache over the preceding two weeks. She had a new diagnosis of diabetes mellitus on admission. Computed tomography scan of the thorax, abdomen, and pelvis showed bilateral pneumonia with liver abscesses. The patient was treated with empirical broad-spectrum antibiotics before *K. pneumoniae* was isolated from cerebrospinal fluid and blood cultures. The isolate was further characterised as a ST23 (ST: sequence type), serotype K1 hypervirulent strain using Nanopore sequencing. Despite admission to the intensive care unit, the patient died within 48 hrs of admission.

**Conclusions:**

This case demonstrates the need for increased awareness of hypervirulent *K. pneumoniae*, even in countries where it occurs infrequently. Novel, rapid, sequencing technologies can support diagnosis in unusual presentations.

## 1. Background

Classical *Klebsiella pneumoniae* (cKp) is an opportunistic pathogen that typically causes urinary tract infections and is a leading cause of nosocomial pneumonia. cKp infections are most common amongst those at the extremes of age or in those who are immunosuppressed. In contrast, hypervirulent *K. pneumoniae* (hvKp) causes severe disseminated infections in otherwise healthy individuals [1]. hvKp is associated with pyogenic liver abscesses as well as pneumonia, meningitis, and endophthalmitis. The strain is rarely seen in Northern Europe, with only occasional case reports in the literature; however, colonisation studies have found hvKp in 0.0–16.7% of community members in South-East Asian countries [2–6]. Although hvKp has been associated with increased viscosity of colonies on agar plate, there is no reliable way to distinguish hvKp from cKp phenotypically and so a high degree of clinical suspicion is required for rapid diagnosis and optimised treatment.

The decreasing cost and size of portable sequencing devices are of increasing interest in atypical clinical presentations of infectious diseases to rapidly genotype pathogens. Having this information available routinely in clinical microbiology laboratories could aid diagnosis and ensure rapid treatment.

We report the case of a woman with hepatic abscesses, bilateral pneumonia, and meningitis with *K. pneumoniae* grown on blood and cerebrospinal fluid culture.

The infection was unfortunately fatal, and subsequent sequencing using a portable nanopore-based device further characterised the isolate as a hypervirulent strain.

## 2. Case Presentation

A female patient in her 60s presented to a hospital emergency department with a 24-hour history of vomiting. She had a two-week history of right upper quadrant abdominal pain and severe frontal headache. She did not report diarrhoea or weight loss and had no history of neck stiffness, photophobia, rash, or limb weakness. She had been generally well since arriving in the United Kingdom (UK) from China 18 months ago. She had no relevant medical history of chronic disease and she was not formally employed. No other family members had been unwell recently, and she reported no foreign travel since arriving in the UK.

Blood tests on admission showed a mild leucocytosis, with a white blood cell (WBC) count of 10.6 × 10^9^/L (neutrophils 9.5 × 10^9^/L, lymphocytes 0.7 × 10^9^/L, and eosinophils 0.1 × 10^9^/L), and severe thrombocytopaenia (platelets 17 × 10^9^/L). Liver function tests were deranged with increased bilirubin 21 *μ*mol/L, alanine aminotransferase 169 IU/L, and alkaline phosphatase 337 IU/L. Coagulation was abnormal with a prothrombin time of 10.4 seconds and a fibrinogen level >9.3 g/L. Other biochemical tests were unremarkable, with normal sodium (140 mmol/L), potassium (3.1 mmol/L), and creatinine (63 *μ*mol/L) and a mildly raised urea (9.3 mmol/L). The C-reactive protein concentration was very high at 250.6 mg/L. A urine dipstick was negative for leucocytes, nitrites, and blood, but showed high (2+) glucose and very high (3+) ketones.

The patient was treated as presumed biliary sepsis with intravenous amoxicillin and clavulanic acid (co-amoxiclav; 1.2 g intravenous three times daily). She was admitted to the acute medical unit and blood was sent for microbial culture. An ultrasound of the abdomen showed multiple solid, mixed echogenicity lesions in both lobes of the liver, the largest of which measured 41 × 34 mm, a normal gallbladder, and nondilated common bile duct. A computed tomography (CT) scan of the brain was reported as normal.

**Figure 1:**
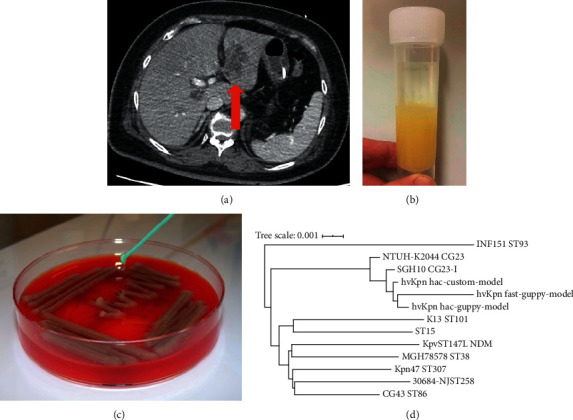
(a) Computed tomography scan of the abdomen showing multiple liver abscesses. (b) Universal container with turbid cerebrospinal fluid. (c) Blood agar plate with *Klebsiella pneumoniae* culture. (d) Genome distance tree comparing the clinical hypervirulent *K. pneumoniae* isolate with 10 reference *K. pneumoniae* isolates produced using Mash.

Twelve hours after admission, the patient became less responsive with a Glasgow Coma Scale 7/15 (E2 V2 M3). Venous blood gas showed metabolic acidosis (pH 7.12; HCO_3_ 8 mmol/L), hyperglycaemia (glucose 29.1 mmol/L), and marked ketosis (ketones 7.0 mmol/L). She was intubated for airway protection and started on an intravenous insulin and fluid resuscitation regime for diabetic ketoacidosis. She was given empirical treatment for presumed central nervous system infection with cefotaxime 2 g IV four times daily and aciclovir 10 mg/kg IV three times daily. A repeat CT scan of the brain was reported as normal, and she was admitted to the intensive care unit (ICU). A CT scan of the chest, abdomen, and pelvis showed bilateral pneumonia, appearances suggestive of an abscess within the left lobe of the liver, and moderate intra-abdominal free fluid (Figure 1(a)). A blood film showed marked thrombocytopaenia with no fragments or clots. One pool of platelets was transfused in order to enable a lumbar puncture to be performed to collect cerebrospinal fluid (CSF).

The CSF was turbid and yellow (Figure 1(b)), with a very high opening pressure of >57 centimetres of CSF (above the maximum recordable level of the manometer). Both the CSF protein and WBC count were markedly elevated at 11.8 g/L and 50,000/m^3^ (85% polymorphs and 15% lymphocytes), respectively. The CSF glucose was low at 1.0 mmol/L, and the red blood cell count was 640 cells/mm^3^. The sample was positive for xanthochromia, but no organisms were seen on Gram stain or acid-fast stain. Blood and CSF cultures subsequently grew *K. pneumoniae*, which was reported as sensitive to cefotaxime and gentamicin. The CSF was PCR negative for *M. tuberculosis*, herpes simplex DNA, Zika virus RNA, and enterovirus RNA. A blood HIV test was negative. The HbA1c level was 100 mmol/mol, suggestive of persistently high blood glucose levels in the preceding months, consistent with a new diagnosis of diabetes mellitus. Following admission to the ICU, the patient was found to have fixed and dilated pupils and, following brainstem testing, a decision was made to withdraw care. The patient died 18 hours later.

The blood culture isolate was cultured onto Columbia Blood Agar (Oxoid, UK) and incubated overnight at 37°C. A single colony was picked and subcultured overnight. The antimicrobial susceptibility of the isolate was determined using the N350 card on the VITEK2^®^ instrument (bioMeriéux, France). DNA was extracted and sequenced on a MinION (Oxford Nanopore Technologies); details are shown in Supplemental Material.

Basecalling was performed using Guppy v3.4.4 with three approaches: a fast option with default settings, a high-accuracy option with a methylation-aware model, and a high-accuracy option with a custom model trained on *Klebsiella* genomes. All read sets were trimmed, filtered, and assembled using Flye v2.7 [7], and draft assemblies were further polished with Racon v1.3.1 [8] and Medaka v 0.7.1 to generate final consensus assemblies (available in FigShare, doi: 10.6084/m9.figshare.11770908; see details in Supplemental Material).

Species confirmation and *Klebsiella*-specific genotyping including multilocus sequence typing (MLST), AMR, and virulence determinants were performed using Pathogenwatch (https://pathogen.watch), Kaptive (https://github.com/katholt/Kaptive), and Kleborate (https://github.com/katholt/Kleborate) [9]. Reference genomes of common hypervirulent or antimicrobial-resistant K. *pneumoniae* (Supplementary Table S1) were downloaded from NCBI and compared to the consensus assemblies by estimating pairwise genome distances using Mash [10] and then constructing a neighbour-joining tree in R.

The blood culture isolate was hypermucoid (Figure 1(c)) and identified by matrix-assisted laser desorption/ionization time-of-flight mass spectrometry (MALDI-TOF MS) as *K. pneumoniae*. It was resistant to ampicillin but was susceptible to all other antimicrobials tested. Sequencing via MinION yielded (after basecalling and demultiplexing) ∼2 million high-quality reads totalling ∼6 Gbp, giving ∼1150-fold coverage of the genome. Average read length was 3.2 Kbp, maximum read length 70.6 Kbp, and N50 6.0 Kbp. All three assemblies resolved two circular molecules: a 5.4 Mbp chromosome and a 228 Kbp plasmid.

The genome sequence confirmed the species identification as *K. pneumoniae*. Distance-based phylogenetic analysis showed the novel isolate clustered with the ST23 (sequence type) K1 hypervirulent clinical isolates SGH10 [11] and NTUH-K2044 [12] and was closest to SGH10 which is representative of the dominant globally distributed liver abscess-associated subclade CG23-I (Figure 1(d)). Genotyping analysis of nanopore-only data is complicated by basecalling error rates >0.1% [13]. Nevertheless, the genome sequence assembled from reads basecalled with a *K. pneumoniae*-trained custom model was identified as a single-locus variant of ST23, with virulence-associated features typical of CG23-I [14]: capsular serotype K1, the genotoxin colibactin (clb/pks), acquired siderophore systems yersiniabactin (ybt), aerobactin (iuc1), and salmochelin (iro1), plus rmpA and rmpA2 (associated with hypermucoidy). The assembly derived from default basecalling identified the presence of the loci but yielded less precise genotypes (see Supplementary Table S2). The iuc1, iro1, rmpA, and rmpA2 were located in the 228  plasmid, which showed full-length homology with pSGH10, a type I *K. pneumoniae* virulence plasmid (KpVP-1) typical of ST23 [9].

## 3. Discussion and Conclusions

We present the case of a patient with disseminated hvKp infection. hvKp causes disseminated disease and needs to be rapidly identified in clinical cases to prevent the devastating consequences of overwhelming infection. Rapid sequencing available via nanopore technology such as the MinION device enables prompt identification of pathogenic strains and allows clinicians to appropriately investigate and treat patients. The patient in this case had risk factors for hvKp, including Chinese origin, but had been residing in the UK for 18 months and had been apparently well prior to presentation. Infections caused by hvKp strains were initially reported from South-East Asia in the 1980s but have spread worldwide [15]. Diabetes mellitus is another well-recognised risk factor for *K. pneumoniae* liver abscess [16]; although the patient was not known to be diabetic prior to admission, she presented in diabetic ketoacidosis, with evidence of long-term poor glycaemic control.

We used a rapid sequencing platform to analyse the bacterial isolate and identified it as *K. pneumoniae* of capsular serotype K1 and sequence type ST23, which accounts for the majority of *K. pneumoniae* liver abscess cases and is widespread in South-East Asia. ST23 strains with the K1 capsule type are especially prevalent in China [17] and are those most commonly associated with progression to central nervous system infection [18]. The genome sequence was closely related to two previously reported ST23 liver abscess isolates, SGH10 (from a case in Singapore) [11] and NTUH-K2044 (from a patient in Taiwan with liver abscesses and meningitis) [12]. The isolate carried all the acquired virulence factors typical of ST23, including the virulence plasmid and chromosomally encoded colibactin (a marker of the major pyogenic liver abscess-associated sublineage, CG23-I) [11].

This work shows how the portable MinION device can be used to rapidly clarify the aetiology of unusual case presentations, in this case confirming that the infection was caused by a previously recognised hvKp strain with known virulence factors, and to rule out a novel emerging hypervirulent strain. However, there remain technical issues and limitations; the high-accuracy basecalling required for accurate genotyping takes substantial computing resources and clock time (≥24 hours), and best results were achieved with a model that had been previously custom-trained on *K. pneumoniae* [13]. Once a high-quality assembly was achieved, we were able to rapidly extract very informative genotyping using the web-based user-friendly data analysis tool, Pathogenwatch, and to compare the genome to that of previously sequenced isolates.

In summary, this case study demonstrates the benefits of a rapid sequencing method to identify and characterise an unusual, highly pathogenic hvKp strain. Sequencing is becoming easier to perform and less expensive, and bioinformatics analysis tools are simpler to use, heralding the possibility of using this technology in routine clinical microbiology laboratories to rapidly characterise clinically important isolates.

## Data Availability

The sequencing data from this study are available from FigShare, doi: 10.6084/m9.figshare.11770908.

## Supplementary Materials

Supplementary Materials The Supplementary Materials file includes the following information: Sequencing methods and bioinformatic analysis. Table S1: *Klebsiella pneumoniae* isolates included in the phylogenetic tree. Table S2: Kleborate genotyping output for nanopore sequenced *K. pneumoniae* isolate. Supplementary references.
