# Supplementary material for: Rapid Whole Genome Sequencing of Serotype K1 Hypervirulent Klebsiella pneumoniae from an Undocumented Chinese Migrant
Source: Case Rep Infect Dis. 2021 Apr 28;2021:6638780. doi: 10.1155/2021/6638780 (PMC8100418; doi:10.1155/2021/6638780)
Supplement: Supplementary Materials — The Supplementary Materials file includes the following information: Sequencing methods and bioinformatic analysis. Table S1: Klebsiella pneumoniae isolates included in the phylogenetic tree. Table S2: Kleborate genotyping output for nanopore sequenced K. pneumoniae isolate. Supplementary references. [file 6638780.f1.docx]

**SUPPLEMENTAL MATERIAL**

**Sequencing methods and bioinformatic analysis**

Purified colonies were selected for DNA extraction using the QIAamp® DNA mini kit (Qiagen, Germany) following manufacturer’s instructions, with elution in nuclease-free water rather than AE buffer. The eluted DNA was quantified using the Qubit 2.0 fluorometer (Life Technologies, USA). Approximately 400 ng of DNA was taken into library preparation with the Rapid Barcoding Kit (SQK-RBK004) for sequencing on the Oxford Nanopore Technologies (ONT) MinION Mk1B device. The default protocol recommended by ONT was followed for library preparation (Version: RBK_9054_v2_revH_23Jan2018), with 75 µL of the final library loaded onto a MIN-106D R9.4.1 flow cell and sequenced for 48 hours. Basecalling was performed using Guppy v3.4.4 with three approaches: a fast option with default settings, a high-accuracy option with a methylation-aware model, and a high-accuracy option with a custom model trained on *Klebsiella* (1). Basecalled fastq read files had adapters and barcodes removed using Porechop v0.2.4 (<https://github.com/rrwick/Porechop>) and reads were filtered based on quality and read length using Filtlong v0.2.0 (<https://github.com/rrwick/Filtlong>).

All read sets were assembled using Flye v2.7 (2), and draft assemblies were further polished with Racon v1.3.1 (3) and Medaka v 0.7.1 (<https://github.com/nanoporetech/medaka>) to generate final consensus assemblies (available in FigShare, doi: [10.6084/m9.figshare.11770908](https://figshare.com/articles/Assembly_files/11770908)).

**TABLE S1.** *Klebsiella pneumoniae* isolates included in the phylogenetic tree

| **Isolate name in tree** | **Alternative isolate name** | **Reference** | **Sequence data location** |
| --- | --- | --- | --- |
| hvKp isolate | n/a | This study | [SRR11050860](https://trace.ncbi.nlm.nih.gov/Traces/sra/?run=SRR11050860) |
| NTUH-K2044 CG23 | n/a | Wu, Li (4){Wu, 2009 #328} | GCF_000009885.1 |
| SGH10 CG23-I | n/a | Lam, Wyres (5) | [GCF_002813595.1](https://www.ncbi.nlm.nih.gov/assembly/GCF_002813595.1) |
| ST258 | 30684/NJST258 | Deleo, Chen (6) | [GCF_000597905.1](https://www.ncbi.nlm.nih.gov/assembly/GCF_000597905.1) |
| ST15 | KP_NORM_BLD_2015_112126 | Lam, Wyres (7) | [GCF_003855335.1](https://www.ncbi.nlm.nih.gov/assembly/GCF_003855335.1) |
| ST147L | KpvST147L_NDM | Turton, Payne (8) | [GCF_002087215.1](https://www.ncbi.nlm.nih.gov/assembly/GCF_002087215.1) |
| ST38 | MGH78578 | McClelland, Sanderson (9) | [GCF_000016305.1](https://www.ncbi.nlm.nih.gov/assembly/GCF_000016305.1/) |
| ST307 | Kpn47 | Domokos, Damjanova (10) | [GCF_004323145.1](https://www.ncbi.nlm.nih.gov/assembly/GCF_004323145.1) |
| ST93 | INF151 | Wyres, Wick (11) | [QWFT01000000](https://www.ncbi.nlm.nih.gov/nuccore/QWFT00000000.1) |
| ST86 | CG43 | Lin, Lo (12) | [GCF_000474015.1](https://www.ncbi.nlm.nih.gov/assembly/GCF_000474015.1) |
| ST101 | K13 | Avgoulea, Di Pilato (13) | [GCF_003034525.1](https://www.ncbi.nlm.nih.gov/assembly/GCF_003034525.1) |

**TABLE S2.** Kleborate genotyping output for genome assemblies constructed from three alternatively base-called read sets, generated from the same set of raw signal data by sequencing the novel *K. pneumoniae* isolate on a MinION (ONT).

| **Assembly** | **fast-guppy-model** | **hac-guppy-model** | **hac-custom-model** |
| --- | --- | --- | --- |
| **species** | *Klebsiella pneumoniae* | *Klebsiella pneumoniae* | *Klebsiella pneumoniae* |
| **ST** | 0 | ST23-2LV | ST23-1LV |
| **virulence_score** | 5 | 5 | 5 |
| **resistance_score** | 0 | 0 | 0 |
| **Yersiniabactin** | ybt unknown | ybt unknown | ybt unknown |
| **YbST** | 0 | 0 | 0 |
| **Colibactin** | clb unknown | clb unknown | clb unknown |
| **CbST** | 0 | 0 | 0 |
| **Aerobactin** | iuc unknown | iuc 1 | iuc 1 |
| **AbST** | 0 | 16-3LV | 37-1LV |
| **Salmochelin** | iro unknown | iro unknown | iro 1 |
| **SmST** | 0 | 0 | 2-1LV |
| **rmpA** | rmpA_5*-54%(KpVP-1) | rmpA_5*-54%(KpVP-1) | rmpA_5*-12%(KpVP-1) |
| **rmpA2** | rmpA2_2*-75% | rmpA2_8*-54% | rmpA2_8*-55% |
| **wzi** | - | - | - |
| **K_locus** | KL1 | KL107 | KL1 |
| **K_locus_confidence** | None  (100% coverage,  99.603% identity to KL1) | None  (40% coverage*,  99.880% identity to KL107) | None  (100% coverage  99.888% identity to KL1) |
| **O_locus** | O1/O2v2 | O1v2 | O1v2 |
| **O_locus_confidence** | None | None | None |
| **AGly** | - | - | - |
| **Col** | MgrB-87%;PmrB-10% | PmrB-42% | - |
| **Fcyn** | - | - | - |
| **Flq** | - | - | - |
| **Gly** | - | - | - |
| **MLS** | - | - | - |
| **Ntmdz** | - | - | - |
| **Phe** | - | - | - |
| **Rif** | - | - | - |
| **Sul** | - | - | - |
| **Tet** | - | - | - |
| **Tgc** | - | - | - |
| **Tmt** | - | - | - |
| **Omp** | OmpK35-90%;OmpK36-85% | - | OmpK35-72%;OmpK36-66% |
| **Bla** | SHV-190* | - | - |
| **Bla_Carb** | - | - | - |
| **Bla_ESBL** | - | - | - |
| **Bla_ESBL_inhR** | - | - | - |
| **Bla_broad** | - | SHV-11^ | SHV-11^ |
| **Bla_broad_inhR** | - | - | - |

*Note the low coverage of the capsule (K) locus in this assembly appears to be because the chromosome contig happens to end in the middle of the K locus, and the assembler Flye sometimes loses sequence off the end of the assembly (1).

**Supplementary References**

1. Wick RR, Holt KE. Benchmarking of long-read assemblers for prokaryote whole genome sequencing. F1000Res. 2019;8:2138.

2. Kolmogorov M, Yuan J, Lin Y, Pevzner PA. Assembly of long, error-prone reads using repeat graphs. Nat Biotechnol. 2019;37:540-46.

3. Vaser R, Sovic I, Nagarajan N, Sikic M. Fast and accurate de novo genome assembly from long uncorrected reads. Genome Res. 2017;27(5):737-46.

4. Wu K-M, Li L-H, Yan J-J, Tsao N, Liao T-L, Tsai H-C, et al. Genome sequencing and comparative analysis of Klebsiella pneumoniae NTUH-K2044, a strain causing liver abscess and meningitis. Journal of bacteriology. 2009;191(14):4492-501.

5. Lam MMC, Wyres KL, Duchene S, Wick RR, Judd LM, Gan YH, et al. Population genomics of hypervirulent Klebsiella pneumoniae clonal-group 23 reveals early emergence and rapid global dissemination. Nat Commun. 2018;9(1):2703.

6. Deleo FR, Chen L, Porcella SF, Martens CA, Kobayashi SD, Porter AR, et al. Molecular dissection of the evolution of carbapenem-resistant multilocus sequence type 258 Klebsiella pneumoniae. P Natl Acad Sci USA. 2014;111(13):4988-93.

7. Lam MMC, Wyres KL, Wick RR, Judd LM, Fostervold A, Holt KE, et al. Convergence of virulence and MDR in a single plasmid vector in MDR Klebsiella pneumoniae ST15. J Antimicrob Chemother. 2019.

8. Turton JF, Payne Z, Coward A, Hopkins KL, Turton JA, Doumith M, et al. Virulence genes in isolates of Klebsiella pneumoniae from the UK during 2016, including among carbapenemase gene-positive hypervirulent K1-ST23 and 'non-hypervirulent' types ST147, ST15 and ST383. J Med Microbiol. 2018;67(1):118-28.

9. McClelland M, Sanderson KE, Spieth J, Clifton SW, Latreille P, Courtney L, et al. Complete genome sequence of Salmonella enterica serovar Typhimurium LT2. Nature. 2001;413(6858):852-6.

10. Domokos J, Damjanova I, Kristof K, Ligeti B, Kocsis B, Szabo D. Multiple Benefits of Plasmid-Mediated Quinolone Resistance Determinants in Klebsiella pneumoniae ST11 High-Risk Clone and Recently Emerging ST307 Clone. Front Microbiol. 2019;10:157.

11. Wyres KL, Wick RR, Gorrie C, Jenney A, Follador R, Thomson NR, et al. Identification of Klebsiella capsule synthesis loci from whole genome data. Microb Genom. 2016;2(12):e000102.

12. Lin FY, Lo YL, Tang CY, Chen YT, Lu CW, Wang WC, et al. Complete genome sequence of Klebsiella pneumoniae CG43, a K2 serotype liver abscess isolate. Submitted to the EMBL/GenBank/DDBJ databases. 2013.

13. Avgoulea K, Di Pilato V, Zarkotou O, Sennati S, Politi L, Cannatelli A, et al. Characterization of Extensively Drug-Resistant or Pandrug-Resistant Sequence Type 147 and 101 OXA-48-Producing Klebsiella pneumoniae Causing Bloodstream Infections in Patients in an Intensive Care Unit. Antimicrob Agents Chemother. 2018;62(7).
